# Number of teeth and functional disability in community‐dwelling older adults

**DOI:** 10.1111/ger.12775

**Published:** 2024-07-10

**Authors:** Takamasa Komiyama, Takashi Ohi, Yoshitada Miyoshi, Mana Kogure, Naoki Nakaya, Atsushi Hozawa, Ichiro Tsuji, Makoto Watanabe, Yoshinori Hattori

**Affiliations:** ^1^ Division of Aging and Geriatric Dentistry, Department of Rehabilitation Dentistry Tohoku University Graduate School of Dentistry Miyagi Japan; ^2^ Japanese Red Cross Ishinomaki Hospital Ishinomaki Miyagi Japan; ^3^ Department of Preventive Medicine and Epidemiology, Tohoku Medical Megabank Organization Tohoku University Sendai Miyagi Japan; ^4^ Division of Epidemiology, Department of Health Informatics and Public Health Tohoku University School of Public Health, Graduate School of Medicine Sendai Miyagi Japan; ^5^ Institute of Living and Environmental Sciences Miyagi Gakuin Women's University Sendai Miyagi Japan

**Keywords:** disability, older adult, oral health, population attributable fraction, tooth loss

## Abstract

**Introduction:**

This study determined whether tooth loss was associated with the development of functional disability and estimated the population attributable fraction (PAF) of functional disability due to tooth loss, along with risk factors for functional disability such as physical function and cognitive impairment.

**Methods:**

The participants were 838 community‐dwelling older adults aged ≥70 years living in the Tsurugaya district in Japan in 2003. The exposure variable was the number of remaining teeth (counted by trained dentists). Other variables were age, sex, depressive symptoms, cognitive impairment, educational attainment, physical function and social support. The Cox proportional hazards model was applied to estimate hazard ratios (HRs) and 95% confidence intervals (CIs) of the incidence of functional disability for each risk factor, such as tooth loss. The functional disability PAF due to tooth loss was estimated, and risk factors for functional disability were identified.

**Results:**

In total, 619 (73.9%) participants developed functional disability during follow‐up. A multivariable model showed that those with <20 teeth (HR, 1.28; 95% CI, 1.08–1.53) were more likely to develop functional disability than those with 20 teeth or more. PAF estimation for functional disability was shown to have decreasing values in the following order: age, female sex, tooth loss and reduced physical function.

**Conclusions:**

Tooth loss was associated with the development of functional disability in community‐dwelling older Japanese adults. While retaining teeth may be a potential strategy for avoiding functional disability, clinical studies on the effect of dental treatment on preventing functional disability are warranted.

## 1 INTRODUCTION

Poor oral health in older adults, especially conditions such as tooth loss, is a public health problem that needs to be addressed.^1^ Data from the United States have shown that the prevalence of adults (aged ≥65 years) with edentulism is 17.3%.^2^ According to the Survey of Dental Diseases in Japan, the prevalence of <20 teeth in adults in the age groups 65–74 years and 75 years or over is estimated to be 31.1% and 53.9%, respectively.^3^ Besides this widespread tooth loss in older adults, several reports have shown that poor oral health, including tooth loss, is associated with poor general health conditions, such as frailty,^4^, ^5^, ^6^ dementia,^7^ and mortality.^8^, ^9^ Moreover, poor oral health is associated with functional disability,^10^, ^11^, ^12^, ^13^ which in turn is associated with medical care burden and is a major public health issue,^14^ thus warranting identification of modifiable factors.

The population attributable fraction (PAF) estimates the proportion of disease cases (which is desirable to be determined using a prospective cohort study) that would not occur in a population if an individual risk factor was eliminated.^15^ Recent research has shown that oral health, especially tooth loss, is associated with mortality, showing a PAF.^16^ As tooth loss in older age has a high prevalence and the association between oral health and functional disability is suggested to share the mechanism with the association between oral health and mortality, it may be that tooth loss is associated with the incidence of functional disability.

This prospective cohort study aimed to examine the extent to which tooth loss is associated with functional disability by calculating the PAF for functional disability and showing this with risk factors for functional disability, such as age, physical function and cognitive function. We hypothesised that tooth loss would be positively associated with functional disability incidence, but that its PAF would be lower than those for age and physical function.

## 2 MATERIALS AND METHODS

### 2.1 Study design

This prospective cohort study is a part of the Tsurugaya Project conducted in 2002 and 2003; the present study included participants from the 2003 survey. In 2003, the project conducted a comprehensive geriatric assessment (CGA) of community‐dwelling older persons living in the Tsurugaya district, a suburban area of Sendai City in northern Japan. Included in the study were all community‐dwelling older adults aged ≥70 years living in the Tsurugaya district in 2003; they were invited by mail to participate in the project, and they were assessed at the examination venue. The project provided a transportation service if appropriate. Our exposure was the number of remaining teeth, which was counted by trained dentists. The primary outcome was the incidence of functional disability, which was determined as the first certification of long‐term care insurance in Japan. It was collected until June 2020.

### 2.2 Study sample

**FIGURE 1:**
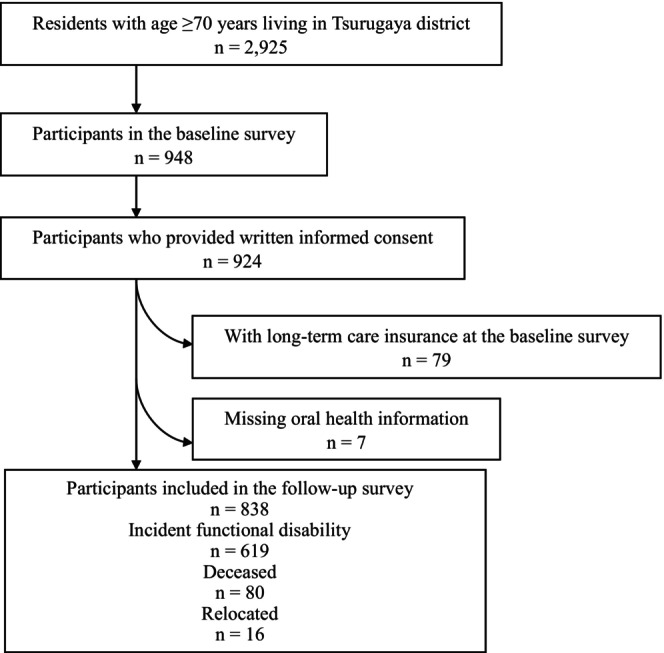
Flow diagram of the participant selection for the study.

The detailed study protocol for the Tsurugaya Project has been described previously.^17^, ^18^ During the CGA, a dental examination was conducted, and the participants answered a questionnaire regarding oral health behaviour and awareness.^19^ Figure 1 shows the flow diagram of the selection of study participants. Briefly, 2925 adults living in the Tsurugaya district aged ≥70 years as of March 2003 were invited to the CGA. Of these, 948 (32.4%) participated in the baseline survey conducted in July 2003, and 924 of these respondents agreed to participate in this study. In the present study, 79 participants who were already certified by long‐term care insurance (LTCI) were excluded, along with 7 participants who did not have data on the number of remaining teeth. The final analysis included 838 participants.

### 2.3 Dental examination

Five trained dentists counted the number of remaining teeth (which was the total number of teeth in the oral cavity) for each participant. Residual roots were not included in the number of remaining teeth.

### 2.4 Other measurements

Participants completed a questionnaire in the 2003 survey that included questions on age, sex, smoking, alcohol consumption and educational attainment. The 30‐item Geriatric Depression Scale was used as an indicator of depressive symptoms,^20^ and participants were deemed to have depressive symptoms if they scored ≥11 points. The Mini‐Mental State Examination (MMSE) was used to measure cognitive impairment,^21^ and participants with MMSE scores ≥26 and <26 were considered to have normal cognition and slight cognitive impairment, respectively.^19^ The Timed‐Up‐and‐Go (TUG) test, which measures the time (in seconds) needed by participants to rise from a standard armchair, walk straight for 3 m, turn around, walk back to the chair and squat down, was used to evaluate physical function.^22^ A duration of ≥11 s indicates reduced physical function, and this cut‐off value was applied for musculoskeletal ambulation disability symptoms, which are related to functional disability.^23^ Five questions were asked to determine social support: (1) Do you have someone whom you can consult when you are in trouble? (2) Do you have someone whom you can consult when your physical condition is poor? (3) Do you have someone who can help with your daily housework? (4) Do you have someone who can take you to a hospital when your physical condition is not good? (5) Do you have someone who can take care of you when you are ill and bedridden? Based on their answers, participants were classified as having restricted (responding “no” to at least one question) or unrestricted (responding “yes” to all questions) social support.^20^

### 2.5 Follow‐up and outcome

The primary outcome variable for this study was the onset of functional disability, defined by the first certification of LTCI in Japan. This criterion has been adopted in several previous epidemiological studies.^11^, ^24^, ^25^ The detailed LTCI system has been described previously.^26^ Briefly, every resident ≥40 years old pays an insurance premium, and every person ≥65 years old is eligible to receive formal caregiving services depending on the level, with support levels ranging from 1 to 2 and care levels ranging from 1 to 5. Eligibility for the LTCI certification is determined upon application of older individuals or their caregivers through (a) an on‐site assessment of the current physical and mental status of individuals and their utilisation of medical resources, using a questionnaire developed by the Ministry of Health, Labor and Welfare and (b) a subsequent needs assessment performed according to the written opinion of the attending physician, following which, a municipal certification committee decides on whether the individual should receive the insurance benefit and the grade.^27^

The researchers obtained data on LTCI certification and its date from the Sendai Municipal Authority, which included information on care levels and dates of relocation from Sendai City or death. The observation period ranged from the baseline survey in 2003 until either the approval date for LTCI certification or June 30, 2020. Censored cases consisted of participants who had relocated from Sendai or who had died.

### 2.6 Data analysis

The baseline characteristics of the study participants in 2003 were evaluated in accordance with the presence or absence of functional disability using Fisher's exact test. Hazard ratios (HRs) and 95% confidence intervals (CIs) for the incidence of functional disability were calculated using the Cox proportional hazards model. Two models were established, with model 1 being the null model. The following factors were adjusted in the multivariable model: age (70–74, 75–79, 80–84 or ≥85 years), sex (male or female), smoking status (never, former or current), alcohol consumption (never, former or current), educational attainment (<18 or ≥18 years), depressive symptoms (Geriatric Depression Scale score <11 or ≥11), cognitive impairment (MMSE score <26 or ≥26), physical function (TUG score ≥11 or <11) and social support (yes or no) (model 2). PAFs were estimated for the onset of functional disability due to each risk factor. After multiple Cox proportional hazards model analyses, all PAFs and 95% CIs were calculated using the “punafcc” package.

Two sensitivity analyses were performed. First, the indicator of physical function was changed from the TUG to the six‐item Physical Function Scale of the Short‐Form General Health Survey adapted from the Medical Outcome Study (MOS).^28^ According to the MOS score, participants were classified into two groups: those capable of performing vigorous physical activity (scores 5 or 6) and those capable of performing moderate or low physical activity (scores 0–4).^29^ Second, age was treated as a continuous variable; thus, age was eliminated from the estimation of PAF. Third, the cut‐off for the number of teeth was changed to 21 based on previous research,^10^ and the number of teeth was treated as a continuous variable to further examine the association between tooth loss and functional disability.

All analyses used Stata version 17.0 (Stata Corp, College Station, TX, USA). For all tests, statistical significance was set at *α* = .05. Missing data were recorded into separate “missing” categories to maximise the number of participants included in the analysis and thereby maximise statistical power.

## 3 RESULTS

The follow‐up comprised 7616 person‐years (average, 9.1 years; minimum, .1 years; maximum, 17.0 years), and 619 (73.9%) participants developed a functional disability (with an average of 8.0 years until that point; standard deviation, 4.5 years).

Table 1 shows functional disability incidence by baseline characteristics. Variables that differed significantly in functional disability incidence were age, sex, alcohol consumption, educational attainment, physical function, social support and the number of remaining teeth.

**TABLE 1:** Baseline characteristics according to functional disability.

Variable	Overall	Functional disability		*p*‐value	Cases/1000 person‐years
		Incidence (*n* = 619)	No incidence (*n* = 219)		
Age					
70–74 years	448	286	162	<.001	58.1
75–79 years	250	206	44		105.7
80–84 years	105	96	9		170.4
≥85 years	35	31	4		241.9
Sex					
Male	404	273	131	<.001	70.1
Female	434	346	88		93.0
Smoking					
Never	469	359	110	.224	83.9
Former	270	189	81		75.0
Current	86	61	24		86.6
Drinking					
Never	319	241	78	.044	84.6
Former	87	58	29		78.8
Current	360	259	101		74.2
Depressive symptoms	207	165	42	.068	129.1
Cognitive impairment	76	59	17	.773	119.8
<18 years of educational attainment	278	218	60	.037	94.8
Reduced physical function	123	107	16	<.001	158.9
Restricted social support	281	220	61	.003	90.8
Number of remaining teeth					
<20	464	366	98	.001	99.7
≥20	374	253	121		64.1

*Note*: Values are indicated by *n*. *p*‐values were obtained using Fisher's exact test. Smoking, *n* = 825; Drinking, *n* = 766; Depressive symptoms, *n* = 825; Cognitive impairment, *n* = 829; < 18 years of educational attainment, *n* = 821; Reduced physical activity, *n* = 824; Restricted social support, *n* = 792.

Table 2 shows the HRs and 95% CIs for functional disability for each factor. In the multivariate model, those with <20 teeth were more likely to develop functional disability than those with less than 20 teeth (HR, 1.28; 95% CI, 1.08–1.53). Among the other factors, those aged ≥75 years, female, current smoking, cognitive impairment, reduced physical function and restricted social support were more likely to develop functional disability than the participants who belong to each reference group.

**TABLE 2:** The association between each factor and developing functional disability (*n* = 838).

Variable	Model 1 HR (95% CI)	*p*‐value	Model 2 HR (95% CI)	*p*‐value
Age				
70–74 years	Reference		Reference	
75–79 years	2.15 (1.78–2.59)	<.001	1.99 (1.64–2.42)	<.001
80–84 years	3.78 (2.95–4.84)	<.001	3.19 (2.44–4.19)	<.001
≥85 years	7.80 (5.29–11.50)	<.001	6.11 (4.13–9.04)	<.001
Sex				
Male	Reference		Reference	
Female	1.37 (1.17–1.61)	<.001	1.48 (1.15–1.89)	.002
Smoking				
Never	Reference		Reference	
Former	.89 (.74–1.06)	.177	1.25 (.97–1.61)	.088
Current	1.13 (.88–1.45)	.328	1.60 (1.11–2.30)	.011
Drinking				
Never	Reference		Reference	
Former	.95 (.71–1.28)	.740	1.02 (.75–1.39)	.902
Current	.86 (.72–1.02)	.082	1.06 (.85–1.32)	.601
Depressive symptoms				
No	Reference		Reference	
Yes	1.46 (1.21–1.76)	<.001	1.18 (.97–1.45)	.104
Cognitive impairment				
No	Reference		Reference	
Yes	1.74 (1.26–2.41)	.001	1.40 (1.05–1.86)	.022
Educational attainment				
≥18 years	Reference		Reference	
<18 years	1.31 (1.11–1.55)	.001	1.06 (.89–1.27)	.498
Reduced physical function				
No	Reference		Reference	
Yes	2.77 (2.16–3.55)	<.001	1.94 (1.53–2.47)	<.001
Restricted social support				
No	Reference		Reference	
Yes	1.27 (1.07–1.50)	.005	1.21 (1.01–1.44)	.034
Number of remaining teeth				
≥20	Reference		Reference	
<20	1.74 (1.48–2.04)	<.001	1.28 (1.08–1.53)	.005

*Note*: Model 1, null model; model 2 adjusted for all variables.

Abbreviations: CI, confidence interval; HR, hazard ratio.

Table 3 shows the PAFs of functional disability due to each factor. Female sex had the highest PAF value (18.0%), followed by age 70–74 years (16.6%), <20 teeth (13.0%), reduced physical function (8.4%) and restricted social support (6.2%).

**TABLE 3:** PAF of functional disability due to each risk factor.

Variable	Participants (*n*)	PAF^a^ (%)	95% CI
Age			
75–79 years	250	16.6	12.3, 20.6
80–84 years	105	10.7	8.1, 13.1
≥85 years	35	4.2	2.7, 5.7
Female	434	18.0	7.6, 27.3
Current smoking	86	3.7	1.1, 6.3
Current drinking	360	2.4	−6.7, 10.7
Depressive symptom	207	4.1	−.7, 8.7
Cognitive impairment	76	2.7	.4, 4.9
<18 years of educational attainment	278	2.1	−4.1, 8.0
Reduced physical function	123	8.4	5.6, 11.1
Restricted social support	281	6.2	.6, 11.4
With <20 teeth	464	13.0	4.2, 21.1

Abbreviations: CI, confidence interval; PAF, population attributable fraction.

^a^ Positive values indicate factors attributed to functional disability.

In the first sensitivity analysis, the TUG test was replaced with the MOS score as an indicator of physical function. The analysis showed that the HR for developing functional disability was higher among those having ≥20 teeth than those with <20 teeth in model 2 (HR, 1.29; 95% CI, 1.08–1.55; Table S1). Age had the highest PAF value (16.4%; 70–74 years old), followed by female sex (15.7%), <20 teeth (12.8%) and reduced physical function (8.7%) (Table S2). In the second sensitivity analysis, age was used as a continuous variable. The results showed that HR of developing functional disability was higher among those with ≥20 teeth than those with <20 teeth in model 2 (HR, 1.25; 95% CI, 1.05–1.50; Table S3). Female sex had the highest PAF value (17.8%), followed by <20 teeth (11.5%), reduced physical function (8.2%) and restricted social support (6.1%) (Table S4). The HR was higher among those with ≥21 teeth than those with <21 teeth (HR, 1.30; 95% CI, 1.09–1.56) and was lower by one unit decrease in the number of teeth (HR, 1.01; 95% CI, 1.00–1.02; Table S5).

## 4 DISCUSSION

The findings from this prospective cohort study showed that tooth loss was associated with the onset of functional disability up to 17 years later after adjusting for age, sex, depressive symptoms, cognitive impairment, educational attainment, physical function and social support. The estimated PAF for functional disability due to tooth loss was 12.1%, which was less than that of age and female sex—which are non‐modifiable risk factors for functional disability—but not less than the PAF due to physical function, social support and cognitive impairment. These findings are consistent with those of our sensitivity analyses, suggesting that the findings of the primary analysis were reasonably robust.

This study had some limitations. First, the sample size was small; thus, a subgroup analysis, such as one stratified by sex, examining the association between tooth loss and functional disability was not performed. A previous study showed that the degree of contribution to mortality differs between sexes;^16^ thus, further studies are needed to examine the gendered contribution to functional disability. Second, the generalisability of these findings is limited because this study targeted community‐dwelling older Japanese individuals, and the response rate was not high. However, if the participation of older adults with severe tooth loss was limited, there is a possibility that the association between oral health measurements and functional disability was underestimated. Moreover, this study collected oral health information only during the baseline survey, and this prevented consideration of changes in oral health status and receipt of dental treatment during follow‐up. This limitation may have led to misclassification of tooth loss. Future research should investigate the association between oral health and functional disability using data collected at multiple time points. Finally, physical function and cognitive function are important confounding factors in the association between tooth loss and functional disability. While the present study confirmed this association after adjusting for two indicators of physical function (TUG test and MOS) and cognitive function, the findings should be interpreted carefully.

Previous studies have examined the association between oral health and functional disability. In United Kingdom and United States cohorts, tooth loss was associated with activities of daily living and instrumental activities of daily living.^30^ From Japanese cohorts, tooth loss—defined as having <20 teeth—was associated with functional disability, which was defined as the incidence of long‐term care insurance.^31^, ^32^ The findings from the present study were consistent with those of previous studies showing an association between tooth loss and functional disability in prospective cohorts. Regarding the PAF for tooth loss on functional disability, Nakazawa et al.^16^ examined the PAF of mortality due to tooth loss, which has related mechanisms. In that study, tooth loss had a large impact on mortality, especially in men. Although the present study did not stratify by sex because of insufficient sample size, the PAF for tooth loss was consistent with the findings of the previous study.

A suggested pathway based on previous research linking tooth loss with functional disability is via dietary intake and nutritional status. Previous work has shown that multiple tooth loss, which causes masticatory impairment, is associated with the avoidance of fruit and vegetables, resulting in lower intakes of protein, vitamins and dietary fibre.^33^, ^34^ Insufficient dietary intake and malnutrition are associated with functional disability.^35^, ^36^ As another suggested pathway, tooth loss appears to be associated with social isolation;^37^ however, considering that tooth loss has been deemed a multi‐dimensional indicator,^38^ it may be associated with functional disability via a complicated pathway. Otherwise, health awareness and behaviour, including regular dental attendance in childhood, could explain the association.^39^

The PAF scores due for tooth loss were estimated to be similar to physical function. The impact of tooth loss at the individual level was associated less with functional disability than with physical function, which is considered to be a major risk factor for functional disability. Tooth loss at the population level was associated with functional disability because of the larger prevalence of tooth loss; hence, poor oral health (especially tooth loss) in older age can be considered to be a dental public health issue. Further, although severe tooth loss is more commonly observed in the older age groups, these individuals are more likely to have experienced the causes of tooth loss, such as caries and periodontitis, earlier in their lives. Hence, considering that situation, interventions targeting older adults may be too late, and a life‐course approach is required.

The association between tooth loss and functional disability could be explained by factors related to life‐course journeys, such as financial, human and social capital.^40^ Therefore, our findings should be interpreted with caution, although the clinician should be aware that tooth loss could be a factor in preventing functional disability. To confirm the usefulness of retaining teeth in older adults, future research might require conduct an interventional study to determine whether treating oral disease has an effect on adverse health outcomes such as functional disability.

## 5 CONCLUSION

In this 17‐year prospective cohort study, tooth loss, defined as <20 teeth, was associated with functional disability, and PAF of functional disability due to tooth loss and risk factors such as restricted physical function and cognitive impairment were estimated. Although retaining teeth could be a target for preventing functional disability, the evidence regarding clinical‐based data, such as interventional studies on dental treatment and functional disability prevention, would be warranted.

## AUTHOR CONTRIBUTIONS

T.K. analysed the data and wrote the original draft. M.K., N.N., A.H., I.T., M.W. and Y.H. contributed to conceptualisation and methodology. T.O., Y.M., A.H., I.T., M.W. and Y.H. contributed to data collection. All the authors contributed to drafting and revising the manuscript.

## FUNDING INFORMATION

This study was partially supported by a Health Labor Sciences Research Grant (H21‐Choju‐Ippan‐001, H22‐Junkankitou‐Ippan‐001) provided by the Ministry of Health, Labor and Welfare of Japan (Tokyo, Japan) and by JSPS KAKENHI (grant numbers 18 K09674, JP18K09904, 19K19325 and 22K10070). The funding sources had no role in the study design; the collection, analysis or interpretation of the data; the writing of the report; nor in the decision to submit the article for publication.

## CONFLICT OF INTEREST STATEMENT

The authors have no conflicts of interest to declare.

## ETHICS STATEMENT

This study's protocol was reviewed and approved by the Ethics Committee of Tohoku University Graduate School of Medicine (approval number, 2017–1‐312). Written informed consent was obtained from all the participants. All the protocols were followed in accordance with the 1964 Helsinki Declaration and its later amendments.

## Supporting information


Data S1:

## Data Availability

The datasets generated and/or analysed during the current study are not publicly available due to the private information of participants being included in those datasets, but the study datasets are available from the corresponding author upon reasonable request.
