# Supplementary material for: Number of teeth and functional disability in community‐dwelling older adults
Source: Gerodontology. 2024 Jul 10;42(1):86–92. doi: 10.1111/ger.12775 (PMC11870638; doi:10.1111/ger.12775)
Supplement: Supplementary file 1 — Data S1: [file GER-42-86-s001.docx]

**Supplementary materials**

**Supplemental Table 1.** Relationship between each risk factor and the incidence of functional disability when adjusting for MOS in place of TUG

| Variable | Model 1 | | | Model 2 |
| --- | --- | --- | --- | --- |
|  | HR (95% CI) | | | |
| Age |  |  |  | |
| 70–74 years | Reference | | | Reference |
| 75–79 years | 2.15 (1.78–2.59)^**^ | | | 1.97 (1.62–2.40)^**^ |
| 80–84 years | 3.78 (2.95–4.84)^**^ | | | 3.24 (2.46–4.29)^**^ |
| ≥85 years | 7.80 (5.29–11.50)^**^ | | | 5.37 (3.36–8.58)^**^ |
| Sex |  |  |  | |
| Male | Reference | | | Reference |
| Female | 1.37 (1.17–1.61)^**^ | | | 1.39 (1.07–1.80)^**^ |
| Smoking |  | | |  |
| Never | Reference | | | Reference |
| Former | 0.89 (0.74–1.06) | | | 1.22 (0.94–1.58) |
| Current | 1.13 (0.88–1.45) | | | 1.55 (1.08–2.21)^*^ |
| Drinking |  | | |  |
| Never | Reference | | | Reference |
| Former | 0.95 (0.71–1.28) | | | 1.00 (0.72–1.38) |
| Current | 0.86 (0.72–1.02) | | | 1.07 (0.86–1.33) |
| Depressive symptoms |  | | |  |
| No | Reference | | | Reference |
| Yes | 1.46 (1.21–1.76)^**^ | | | 1.16 (0.95–1.43) |
| Cognitive impairment |  | | |  |
| No | Reference | | | Reference |
| Yes | 1.74 (1.26–2.41)^**^ | | | 1.38 (1.02–1.86) |
| Educational attainment |  | | |  |
| ≥18 years | Reference | | | Reference |
| <18 years | 1.31 (1.11–1.55)^**^ | | | 1.10 (0.91–1.32) |
| Reduced physical function | | | |  |
| No | Reference | | | Reference |
| Yes | 1.95 (1.63–2.33)^**^ | | | 1.46 (1.18–1.83)^**^ |
| Restricted social support | | | |  |
| No | Reference | | | Reference |
| Yes | 1.27 (1.07–1.50)^**^ | | | 1.26 (1.05–1.51)^*^ |
| Number of remaining teeth | | | |  |
| ≥20 | Reference | | | Reference |
| 0–19 | 1.70 (1.45–2.00)^**^ | | | 1.29 (1.08–1.55)^**^ |

*P < 0.05; **P < 0.01

Model 1: null model; model 2: adjusted for all variables; HR, hazard ratio; CI, confidence interval; MOS, six-item Physical Function Scale of the Short-Form General Health Survey adapted from the Medical Outcome Study; TUG, Timed-Up-and-Go test.

**Supplemental Table 2.** PAF of risk factors for functional disability when considering MOS in place of TUG

| Variable | Participants (n) | PAF^a^(%) | 95% CI |
| --- | --- | --- | --- |
| Age |  |  |  |
| 75–79 years | 250 | 16.4 | 12.1, 20.5 |
| 80–84 years | 105 | 10.7 | 8.2, 13.2 |
| ≥85 years | 35 | 4.1 | 2.5, 5.6 |
| Female | 434 | 15.7 | 4.3, 25.8 |
| Current smoking | 86 | 3.5 | 0.9, 6.2 |
| Current drinking | 360 | 2.6 | −0.7, 10.9 |
| Depressive symptom | 207 | 3.7 | −1.3, 8.5 |
| Cognitive impairment | 76 | 2.6 | 0.2, 4.9 |
| <18 years of educational attainment | 278 | 3.1 | −3.0, 8.9 |
| Reduced physical function | 208 | 8.7 | 4.0, 13.1 |
| Restricted social support | 281 | 7.4 | 1.8, 12.6 |
| With 0–19 teeth | 464 | 12.8 | 4.1, 20.6 |

^a^Positive values indicate factors attributed to functional disability.

PAF, population attributable fraction; MOS, six-item Physical Function Scale of the Short-Form General Health Survey adapted from the Medical Outcome Study; TUG, Timed-Up-and-Go test.

**Supplemental Table 3.** Relationship between each risk factor and the incidence of functional disability when age was adjusted as a continuous variable

| Variable | Model 1 | | | Model 2 |
| --- | --- | --- | --- | --- |
|  | HR (95% CI) | | | |
| Age (per 1-year older) | 1.15 (1.13–1.17)^**^ | | | 1.13 (1.10–1.15)^**^ |
| Sex |  |  |  | |
| Male | Reference | | | Reference |
| Female | 1.37 (1.17–1.61)^**^ | | | 1.47 (1.14–1.88)^**^ |
| Smoking |  | | |  |
| Never | Reference | | | Reference |
| Former | 0.89 (0.74–1.06) | | | 1.23 (0.95–1.58) |
| Current | 1.13 (0.88–1.45) | | | 1.61 (1.15–2.26)^**^ |
| Drinking |  | | |  |
| Never | Reference | | | Reference |
| Former | 0.95 (0.71–1.28) | | | 0.99 (0.72–1.35) |
| Current | 0.86 (0.72–1.02) | | | 1.05 (0.85–1.31) |
| Depressive symptoms |  | | |  |
| No | Reference | | | Reference |
| Yes | 1.46 (1.21–1.76)^**^ | | | 1.13 (0.92–1.39) |
| Cognitive impairment |  | | |  |
| No | Reference | | | Reference |
| Yes | 1.74 (1.26–2.41)^**^ | | | 1.41 (1.06–1.89) |
| Educational attainment |  | | |  |
| ≥18 years | Reference | | | Reference |
| <18 years | 1.31 (1.11–1.55)^**^ | | | 1.06 (0.89–1.27) |
| Reduced physical function |  | | |  |
| No | Reference | | | Reference |
| Yes | 2.70 (2.11–3.44)^**^ | | | 1.91 (1.51–2.42)^**^ |
| Restricted social support |  | | |  |
| No | Reference | | | Reference |
| Yes | 1.27 (1.07–1.50)^**^ | | | 1.21 (1.01–1.44)^*^ |
| Number of remaining teeth |  | | |  |
| ≥20 | Reference | | | Reference |
| 0–19 | 1.70 (1.45–2.00)^**^ | | | 1.25 (1.05–1.50)^**^ |

*P < 0.05; **P < 0.01

Model 1, null model; model 2: adjusted for all variables; HR, hazard ratio; CI, confidence interval.

**Supplemental Table 4.** PAF of risk factors for functional disability considering age as a continuous variable

| Variable | Participants (n) | PAF^a^(%) | 95% CI |
| --- | --- | --- | --- |
| Female | 434 | 17.8 | 7.2, 27.1 |
| Current smoking | 86 | 3.8 | 1.3, 6.3 |
| Current drinking | 360 | 2.1 | −0.7, 10.4 |
| Depressive symptom | 207 | 3.0 | −2.2, 7.9 |
| Cognitive impairment | 76 | 2.8 | 0.6, 5.0 |
| <18 years of educational attainment | 278 | 2.1 | −4.1, 8.0 |
| Reduced physical function | 123 | 8.2 | 5.4, 11.0 |
| Restricted social support | 281 | 6.1 | 0.5, 11.3 |
| With 0–19 teeth | 464 | 11.5 | 2.7, 19.4 |

^a^Positive values indicate that factors attributed to functional disability.

PAF, population attributable fraction; CI, confidence interval.

**Supplemental Table 5.** The number of teeth and functional disability using different cut-offs and treating the number of teeth as continuous variable

| Variable | Model 1  HR (95% CI) | p-value | Model 2  HR (95% CI) | p-value |
| --- | --- | --- | --- | --- |
|  |  |  |  |  |
| The number of remining teeth |  |  |  |  |
| ≥21 teeth | Reference |  | Reference |  |
| <21 teeth | 1.74 (1.48–2.05) | <.001 | 1.30 (1.09–1.56) | 0.003 |
|  |  |  |  |  |
| The number of remining teeth (continuous: as one decrease) | 1.03 (1.02–1.04) | <.001 | 1.01 (1.00–1.02) | 0.056 |

Model 1, null model; model 2: adjusted for age, sex, smoking, drinking, depressive symptom, cognitive function, educational attainment, physical function, and social support.

Analyses were conducted separately.

HR, hazard ratio; CI, confidence interval.
